# Prevalence of intestinal parasites in a cohort of HIV- infected patients from Antioquia, Colombia

**DOI:** 10.7705/biomedica.5992

**Published:** 2021-10-15

**Authors:** Jorge Botero-Garcés, Esteban Villegas-Arbeláez, Sofía Giraldo, Johanna Urán-Velásquez, Laura Arias-Agudelo, Juan Carlos Alzate-Ángel, Gisela María García-Montoya, Ana Luz Galván-Díaz

**Affiliations:** 1 Unidad de Investigación Clínica, Corporación para Investigaciones Biológicas, Medellín, Colombia Corporación para Investiga. Biológicas Unidad de Investigación Clínica Corporación para Investigaciones Biológicas Medellín Colombia; 2 Grupo de Parasitología, Facultad de Medicina, Corporación Académica para el Estudio de las Patologías Tropicales, Universidad de Antioquia, Medellín, Colombia Universidad de Antioquia Grupo de Parasitología, Facultad de Medicina Corporación Académica para el Estudio de las Patologías Tropicales Universidad de Antioquia Medellín Colombia; 3 Centro Nacional de Secuenciación Genómica, Sede de Investigación Universitaria, Universidad de Antioquia, Medellín, Colombia Universidad de Antioquia Centro Nacional de Secuenciación Genómica Sede de Investigación Universitaria Universidad de Antioquia Medellín Colombia; 4 Unidad de Micología Médica y Experimental, Corporación para Investigaciones Biológicas- Universidad de Santander, Medellín, Colombia. Universidad de Santander Unidad de Micología Médica y Experimental Corporación para Investigaciones Biológicas Universidad de Santander Medellín Colombia; 5 Grupo Pediaciencias, Facultad de Medicina, Universidad de Antioquia, Medellín, Colombia Universidad de Antioquia Grupo Pediaciencias Facultad de Medicina Universidad de Antioquia Medellín Colombia; 6 Grupo de Microbiología Ambiental, Escuela de Microbiología, Universidad de Antioquia, Medellín, Colombia Universidad de Antioquia Grupo de Microbiología Ambiental Escuela de Microbiología Universidad de Antioquia Medellín Colombia

**Keywords:** Intestinal diseases, parasitic, HIV, AIDS-related opportunistic infections, diarrhea, prevalence, Colombia, parasitosis intestinales, VIH, infecciones oportunistas relacionadas con el sida, prevalencia, diarrea, Colombia

## Abstract

**Introduction:**

HIV infection is still a public health problem worldwide and co-infections with other infectious agents including intestinal parasites are of particular concern, mainly in developing countries like Colombia.

**Objective:**

To conduct a cross-sectional study in patients attending an HIV care program in Antioquia given that there have been few intestinal parasites prevalence studies among the HIV population in the country.

**Material and methods:**

We evaluated stool samples from 192 patients by direct wet mount and concentration, modified Ziehl Neelsen staining, and agar plate culture. Univariate and correlation analyses were done to explore the association between socio-demographic and clinical characteristics and parasitological data.

**Results:**

The overall prevalence of intestinal parasites in HIV-positive subjects was 29.2% (56/192; 95% CI: 22.8% - 35.6%). *Entamoeba histolytica/dispar/moshkosvkii* with 13.0% (25/192; 95% CI: 8.2% - 17.8%) and *Blastocystis* with 12.0% (23/192; 95% CI: 7.4% -16.6%) were the most frequent. Opportunistic parasites like *Cryptosporidium* spp. and *Cystoisospora belli* were less prevalent, each one with 0.5% of positive samples (1/192; 95% CI: 0.1% - 1.5%). Commensal protozoa were also detected with a prevalence of 18.8% (36/192; 95% CI: 13.3% - 24.3%). Most of the individuals in the study had a controlled viral load and an LTCD4 count greater than 200 cel/µL. A small percentage (9.3%) had diarrhea. Bivariate analysis and multivariate logistic regression showed that only age and having pets had a significant association with intestinal parasites in this cohort.

**Conclusions:**

Our results confirmed that the evaluated population is at high risk of intestinal parasite infection, which highlights the need for routine screening of gastrointestinal parasites to provide prompt treatment and reduce possible complications.

## Resumen

**Introducción.:**

La infección por HIV y las coinfecciones con otros agentes infecciosos, incluidos los parásitos intestinales, son motivo de especial preocupación en países en desarrollo como Colombia.

**Objetivo.:**

Hacer un estudio transversal en pacientes que asisten a un programa de atención de HIV en el departamento de Antioquia, dado que los estudios de prevalencia de parásitos intestinales en la población con HIV son escasos en el país.

**Materiales y métodos.:**

Se evaluaron 192 muestras de materia fecal mediante examen coprológico directo y por concentración, tinción de Ziehl-Neelsen modificada, y aislamiento en agar. Se hicieron análisis univariados y de correlación, para explorar la asociación entre las características sociodemográficas y clínicas, y los datos parasitológicos.

**Resultados.:**

La prevalencia global de parásitos intestinales en pacientes positivos para VIH fue del 29.2 % (56/192; IC_95%_ 22.8-35.6 %), siendo *Entamoeba histolytica/dispar/moshkosvkii,* con 13.0 % (25/192; IC_95%_ 8.2-17.8 %), y *Blastocystis,* con 12.0 % (23/192; IC_95%_ 7.4-16.6 %), los mas frecuentes. Los parásitos oportunistas Cryptosporidium spp. Y Cystoisospora belli fueron menos prevalentes, cada uno con 0.5 % (1/192; IC_95%_ 0.1-1.5 %) de muestras positivas. También, se detectaron protozoos comensales, con una prevalencia del 18,8 % (36/192; IC_95%_ 13,3-24,3 %). La mayoría de los individuos tenía una carga viral controlada y un recuento de linfocitos T CD4 superior a 200 células/μl. Un pequeño porcentaje (9,3 %) presentó diarrea. La edad y el tener mascotas mostraron una asociación significativa con la presencia de parásitos intestinales.

**Conclusión.:**

Se confirmó que la población evaluada tiene un alto riesgo de infección por parásitos intestinales, lo que resalta la necesidad de un protocolo de diagnóstico para el cribado de dichos agentes, con el fin de brindar un tratamiento rápido y reducir las posibles complicaciones.

The human immunodeficiency virus (HIV) is the etiological agent of acquired immunodeficiency syndrome (AIDS) and causes dysregulation of the immune system killing CD4 T lymphocytes, which predisposes sufferers to opportunistic infections ^1^. Such co-infections are associated with severe clinical symptoms that are usually unnoticed or mild in healthy patients and whose treatment is a challenge due to the difficulties regarding a successful scheme. Co-infections of HIV and parasites including intestinal protozoa and helminths are of particular concern, mainly in low-income areas where they can be major causes of death among AIDS patients ^2^.

Intestinal parasites are still a public health problem worldwide, particularly in third-world countries like Colombia, where inadequate sanitary conditions facilitate their transmission. Several intestinal helminths and protozoa affect humans causing a wide range of symptoms, among them diarrhea, abdominal pain, weight loss, and malnutrition, whose severity also depends on demographic, socio-economic, physiological conditions, and immunological status ^3^.

According to the estimates of the *Fondo Colombiano de Enfermedades de Alto Costo*, there are 109,056 people living with HIV in the country with a prevalence in adults of 0.23%. Despite the advances in antiretroviral therapy and diagnostic techniques, 50% of newly diagnosed people are detected in stage 3 of the infection ^4^. Additionally, the national survey of intestinal parasitism in Colombia, 2012-2014, estimated a national prevalence of soil- transmitted helminths of 29.6%, *Trichuris trichiura* (18.4%) being the most frequent followed by *Ascaris lumbricoides* (11.3%), and hookworms (6.4%). Regarding protozoans, this survey reports a prevalence of 60.3%, *Blastocystis* sp. (57,6%), *Giardia intestinalis* (15,4%), and *Entamoeba histolytica/dispar/ moshkovskii* (12.9%) being the most frequent ^5^. These data confirm the high risk of parasite infection in the Colombian population.

Given the spread of HIV in Colombia, people living with this disease could be coinfected with parasites; however, recent prevalence studies (less than ten years old) among the HIV population are scarce in the country ^6^. In this context, our objective was to determine the prevalence of gastrointestinal parasitic infections among a cohort of HIV patients attending a care program at the *Corporación para Investigaciones Biológicas* (CIB) in Medellín, Colombia. We also evaluated associated socio-demographic factors. This information would widen the knowledge of the epidemiology of parasitic infections in this group of patients.

## Materials and methods

### Characterization of patients and study site

Participants were both male and female with a confirmed HIV/AIDS diagnosis according to the clinical practice guidelines of the Colombian Ministry of Health. They had not used antiparasitic drugs in the previous six months and were followed up at the specialized assistance service (SAE) program for patients living with HIV at CIB in Medellín and other municipalities of Antioquia between May 2018 and April 2019.

### Study design, sample size, and sampling

We conducted a cross-sectional study. The sample size was 222 individuals calculated using the statistical software Epidat 4.0, with a 95% level of confidence (z = 1.96), a confidence interval precision of 5%, and a prevalence of intestinal parasites of 21.4% for HIV patients from Medellín ^7^. Patients were selected from the CIB HIV patient care program database using a simple random sampling technique. The recruitment period went from May 2018 to April 2019.

We weighed the total sample size (222) against the number of patients contributed by subregion (stratum) and then carried out a simple random sampling within each stratum. The patients were randomly selected from the database mentioned above and then invited to meet the researchers to clarify the study objectives and resolve their doubts. Individuals who agreed to participate in the study signed the informed consent and answered a socio-epidemiological survey to gather the data on their gender, age, type of residence, water supply, sewerage system, hand washing, clinical status, CD4+ T-cell count, viral load, Anti-Retroviral Treatment (ART), and HIV infection status following the World Health Organization (WHO) guidelines. Patients were instructed on how to collect their stool samples and received screw-capped containers identified with a sample number.

**Figure 1:**
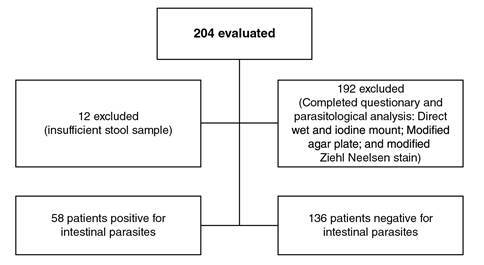
Participation in a survey on HIV and intestinal parasites co-infection in a cohort regularly attending an HIV care program, CIB, Medellín, Colombia

We recruited 204 patients who completed the clinical-epidemiological survey, but only 192 of the samples had the size needed for the parasitological analyses (figure 1). Fecal samples were stored, further processed, and analyzed in the parasitology laboratory at *Universidad de Antioquia*.

### Techniques for the parasitological diagnosis of fecal samples

We collected a stool sample from each participant on the day of their medical appointment. Each fresh stool specimen was processed using the following methods:

1. Direct saline and iodine wet mounts using a commercial sedimentation kit (Mini Parasep SFSolvent Free^™^, Diasys) for the diagnosis of protozoa and helminths; the total amount of sediment obtained in the concentration was evaluated ^8^.
2. Modified agar plate was used for Strongyloides stercoralis detection ^8^, and
3. the Ziehl-Neelsen stain method was used for intestinal apicomplexa detection (*Cryptosporidium* spp., *Cystoisospora belli*, and *Cyclospora cayetanensis*) ^8^

Two stained slides of each sample were prepared and examined by two analysts.

### Statistical analysis

Statistical analyses of the epidemiologic, clinical, and parasitological data were performed using the Statistical Package for the Social Sciences^™^ (SPSS) (version 25, SPSS Inc., Chicago, Illinois) software. Overall socio- demographic and clinical characteristics, as well as specific prevalence, were calculated using descriptive statistics through frequencies and cross-tabulations. The bivariate association of parasitic infections with the independent variables was calculated using t test (student), chi-square, or Fisher’s exact tests, and associations using multivariate logistic regression analysis; 95% confidence intervals (CI) and p<0.05 were considered statistically significant.

### Ethical considerations

The study protocol was approved by the institutional review board of the CIB ethics committee (approval number 19-06-760). Participants were contacted by the researchers who explained the study objectives, procedures, and potential risks. Interested individuals provided written informed consent and, in the case of minors, their parents did. Antiparasitic drugs (albendazole, ivermectin, trimethoprim-sulfamethoxazole, and/or tinidazole) were offered for free to all participants found infected with protozoans and/or helminths through local health care institutions.

## Results

### Baseline characteristics of participants

A total of 204 volunteers were enrolled with a confirmed diagnosis of HIV. Except for one patient, all were receiving some ART regime. The sex ratio (M/F) was approximately 2:1 and 68.6% (95%CI: 62.2% - 75.0%) were men; the average age (± SD) was 48.6 (± 11.7) years ranging from 13 to 80 years; 179 individuals, i.e., 87.7% (179/204; 95%CI: 83.2% - 92.2%) lived in urban areas. Only 9.3% (19/204; 95%CI: 5.3% - 13.3%) of the patients had diarrhea, 13 of them, acute (1-14 days duration), four, chronic diarrhea (> 30 days ), and two, subacute diarrhea (15-30 days). 

Table 1 shows the viral load values and LTCD4+ count. Most of the patients had LTCD4 counts over 200 cel/µL (178/204; 87.3%: 95%CI: 82.7% - 91.9%); we collected stool samples from 94.1% (192/204) of the participants for the parasitological analysis (figure 1).

**Table 1:** Immunological status of the evaluated patients

Immunological status	n (%; 95%CI)
LTCD4+ count	
≤200	26 (12.7; 8.1 to 17.3)
201-499	79 (38.7; 32.0 to 45.4)
≥500	99 (48.6; 41.7 to 55.5)
Total	204 (100)
Viral load	
Controlled (<50 copies/ml)	169 (82.9; 77.7 to 88.1)
Low viremia (50-200 copies/ml)	8 (3.9; 1.2 to 6.6)
Virological failure (>200copies/ml)	27 (13.2; 8.6 to 17.8)
Total	204 (100)

### Prevalence of intestinal parasites

The overall prevalence of intestinal parasites in HIV-positive subjects was 29.2% (56/192; 95%CI: 22.8% - 35.6%). Potentially pathogenic parasites in the cohort (9.4%; 95%CI: 5.3% - 13.5%) were more frequent than commensal parasites (4,7%; 95%CI: 1.7% - 7.7%) while opportunistic parasites were the least common (1,0%; 95%CI: 0.1% - 2.4%). Six different species of potentially pathogenic intestinal parasites were identified with the following order of prevalence: *Entamoeba histolytica/dispar/moshkosvkii,* 13.0% (25/192; 95%CI: 8.2 - 17.8); *Blastocystis,* 12.0% (23/192; 95%CI: 7.4% - 16.6%), and *Cystoisospora belli* (1/192), *Cryptosporidium* spp. (1/192), *Strongyloides stercoralis* (1/192), and *Trichuris trichiura* (1/192), all with 0.5% (95%CI: 0.1% - 1.5%). The global prevalence of commensal parasites was 18.8% (36/192; 95%CI: 13.3% - 24.3%) and only *Entamoeba coli*, *Entamoeba hartmanni*, *Endolimax nana,* and *Iodamoeba butschlii* were found (table 2).

**Table 2:** Frequency of the types of intestinal parasitism

Parasite type	n (%; 95%CI)
Potentially pathogenic and comensal	27 (14.1; 9.2 to 19.0)
Potentially pathogenic only	18 (9.4; 5.3 to 13.5)
Comensal only	9 (4.7; 1.7 to 7.7)
Opportunistic only	2 (1.0; 0.1 to 2.4)
Negative	136 (70.8; 64.4 to 77.2)
Total	192

### Multiparasitism

As mentioned, the prevalence of intestinal parasite infections among the HIV-positive patients was 29.2%. Single-species infections were the most common with 97.4% (187/192; 95%CI: 95.1% - 99.7%) while 2.6% (5/192; 95%CI: 0.35% - 4.9%) were infections by two species: *Entamoeba histolytica/ dispar/moshkosvkii* and *Blastocystis*.

### Socio-demographic and clinical characteristics, environmental variables, and laboratory profile

We analyzed these parameters along with the intestinal parasite prevalence. In the bivariate analysis, no statistically significant association was found with gender, residence, diarrhea, type of diarrhea, LTCD4 count, or viral load category (table 3). Furthermore, there was no evidence of an association between intestinal parasitism and occupation, marital and educational status, the habit of washing before meals, water supply, and feces consistency (data not shown). However, age and having pets showed a significant association (table 3).

**Table 3:** Distribution of socio-demographic, clinical, and immunological parameters according to parasitism

Parameter	Intestinal parasite		DP (95%CI)	^p^
	Positive	Negative		
Patients, n (%): 192 (100)	56 (29.2)	136 (70.8)		
Gender, n (%)				
Male	44 (78.6)	88 (64.7)	-0.8 to 27.5	0.06
Female	12 (21.4)	48 (35.3)		
Residence, n (%)				
Urban	46 (82.1)	122 (89.7)	-37.5 to 8.9	0.150
Rural	10 (17.9)	14 (11.3)		
Pet owners, n (%)				
Yes	33 (60.0)	53 (39.3)	4.6 to 32.1	0.009
No	22 (40.0)	82 (60.7)		
Diarrhea, n (%)				
Yes	7 (14.9)	12 (8.3)	-11.8 to 39.2	0.187
No	40 (85.1)	133 (91.7)		
Diarrhea condition, n (%)				
Acute	8 (88.9)	5 (50.0)	-16.2 to 99.2	0.098
Subacute	1 (11.1)	1 (10.0)		
Chronic	0 (0.0)	4 (40.0)		
Receiving ART, n (%)				
Yes	56 (100)	135 (99,3)	-2.4 to 61.0	0.978
No	0 (0.0)	1 (0.7)		
Mean age (years), X ± SD	52.6 ± 10.0	47,1 ± 10.1	5.6** (2.6 to 9.0)	0.001*
Viral load, X ± SD	230.9 ± 813.1	3394.9 ±16008.7	3164** (-1064.5 to 7392.	4) 0.492*
Viral load category, n (%)				
Controlled	48 (85.7)	112 (82.4)	-25.9 to 19.2	0.851
Low viremia	2 (3.6)	6 (4.4)		
Virological failure	6 (10.7)	18 (13.2)		
LTCD4+ category, n (%)				
≤ 200	2 (4.3)	23 (15.9)	-33.8 to 4.1	0.121
201-499	20 (42.5)	55 (37.9)		
≥ 500	25 (53.2)	67 (46.2)		

DP: difference of proportions

*: t Student test

**: Difference of means

After adjusting for socio-demographic, clinical, and immunological characteristics and potentially confounding variables using multivariate logistic regression, these variables were still significantly associated with intestinal parasitosis. Odds ratio (OR) adjusted by age and the 95% CI were near one ^1^ (table 4).

**Table 4:** Multivariate analysis of the association between socio-demographic, environmental, and clinical parameters in relation to intestinal parasites prevalence

Parameter	β coefficient	OR raw	Adjusted OR	95% CI	p
Age	0.05	-	1.05	1.01 to 1.09	0.008
Sex	0.54	2.0	1.71	0.78 to 3.7	0.18
Residence	0.52	1.8	1.68	0.65 to 4.3	0.281
Having pets	1.07	2.3	2.90	1.4 to 6.0	0.003
Diarrhea	0.61	1.9	1.84	0.64 to 5.3	0.257
LTCD4 category			
≥500		-	-	-	-
200-499	-0.84	1.02	0.43	0.133 to 1.4	0.162
<200	0.21	4.1	1.23	0.60 to 2.5	0.571
Viral load					0.954
Controlled		-	-	-	-
Low viremia	0.08	1.3	1.08	0.23 to 7.2	0.89
Viral failure	0.25	1.2	1.02	0.37 to 3.2	0.772

## Discussion

Intestinal parasitic infections are one of the most common health problems in patients with HIV/AIDS. The immunodeficiency associated with this virus favors the development of moderate to severe, sometimes fatal, infections, which are generally asymptomatic or mild in an immunocompetent host. Recent data on the prevalence of opportunistic/nonopportunistic intestinal parasites in the HIV/AIDS population are scarce in Colombia.

The aim of this work was to study the presence of enteroparasites in a cohort of HIV/AIDS patients from Antioquia including its capital city, Medellín.

The overall prevalence of intestinal parasites (29.2%) was lower than that reported by other authors in the HIV population in Colombia. Flórez, *et al*. ^9^ reported a prevalence of intestinal parasites of 59.1% in HIV-infected patients from Bogotá, and Botero, *et al*., described a prevalence of 32.43% ^3^ and 39.8% ^10^ in immunocompromised individuals (acute and chronic myeloid leukaemia, HIV, and other immunodeficiencies), and HIV patients from Medellín, respectively.

It is important to highlight that most of the patients evaluated in these studies showed intestinal symptoms including chronic diarrhea, which increases the probability of parasite detection; in our cohort, however, only 9.3% of the patients reported this symptom. In the Latin American context, prevalence studies conducted in Brazil have reported HIV patients data that have remained similar in the last 20 years: 28.9% in 1996 ^11^ and 28.88% in 2018 ^12^. Other countries have reported remarkably high parasitic infection rates with prevalences of 67.9% and 73.1% in some areas of Venezuela ^13^ and Perú ^14^, respectively.

Among the intestinal parasites detected in the HIV population in our study, *Entamoeba histolytica/dispar/moshkosvkii* and *Blastocystis* were the most frequent, which agrees with the results obtained by Flórez, *et al*. ^9^ who also described these parasites as the most common in HIV patients from different hospitals in Bogotá, with prevalences of 25.2% and 13% for *Blastocystis* and *E.histolytica/dispar/moshkosvkii*, respectively. Botero, *et al.*^3^^,^^10^ also reported *E.histolytica/dispar/moshkosvkii* as one of the most frequent parasites in immunocompromised patients from Medellín, most of them HIV- positive individuals.

Among the *Entamoeba* species infecting humans, *E. histolytica* is so far the only one associated with amoebiasis*,* one of the most problematic parasitic infections worldwide, particularly in poor communities from developing countries, resulting in severe conditions such as amebic colitis and amebic liver abscess and even in fatal cases ^15^. The impact of HIV infection on amoebiasis occurrence remains controversial. Several studies have shown an increased prevalence of *E. histolytica* infection among HIV-positive patients in México, China, South Africa, and Ethiopia ^16^^-^^19^. However, most of these countries are considered endemic for amoebiasis, i.e., there is a high risk of infection for their population including those with HIV.

*Blastocystis* is one of the most frequent intestinal protists in humans; its prevalence is even higher than that of the other protists commonly reported in man ^20^. In Colombia, the national survey of intestinal parasitism revealed *Blastocystis* as the most prevalent intestinal protist among the children evaluated with 52.1% of positive individuals, which exceeds the prevalence for the *Entamoeba* complex species (*E.histolytica/ dispar/moshkovskii*) with 17% ^5^. Thought to have a commensal relationship with humans initially, the results and observations from different studies have shown that *Blastocystis* is a potentially pathogenic parasite associated with a variety of intestinal symptoms including watery diarrhea, abdominal pain, flatulence, nausea, vomiting, and constipation, among others ^21^. The prevalence of *Blastocystis* infection worldwide is high in HIV/AIDS patients, although data are limited. In a study by Fontanelli, *et al.*^22^, *Blastocystis* infection was common in HIV- positive patients on ART with a prevalence of 25% and homosexual behavior represented a risk factor for its transmission while CD4 count and viremia did not correlate with the presence of this protist. The only symptom associated with *Blastocystis* was flatulence.

Diarrhea caused by opportunistic intestinal protozoa is a common problem in HIV patients with *C. belli* and several species of *Cryptosporidium* among the most frequent pathogens responsible for significant morbidity and mortality in them ^23^^,^^24^. However, in our study, there was a low infection frequency by these protozoa with a 0.5% prevalence for each parasite. Data from Colombia on *Cryptosporidium* prevalence in HIV patients varies ranging from 2.5 to 51.4% ^3^^,^^6^^,^^9^^,^^10^^,^^25^^-^^28^. Although molecular methods (namely PCR) have shown great sensitivity for *Cryptosporidium* detection allowing species identification, most of the studies in the HIV population in Colombia are based on staining techniques. Therefore, the variability in prevalence values in the country may be related more to the clinical, immunological, and virological conditions of the patients than to the diagnostic techniques used. In the case of *C. belli*, studies have registered prevalences between 1.9 and 7.9% in our country ^10^^,^^25^^,^^29^.

Data from a global systematic review and meta-analysis on the opportunistic protozoa infection in HIV patients worldwide showed a prevalence of *Cryptosporidium* ranging from 0 to 78.1% and from 0.2 to 26.9% for *C. belli*^24^. In Latin America and the Caribbean, the estimated pooled prevalence was 13% (95%CI: 7.3-18.7%) and 0.8% (4/436; 95%CI: 0-2.0%) for *Cryptosporidium* spp. and *C. belli*, respectively ^24^. Additionally, a meta-regression analysis showed that patients with diarrhea may be a source of heterogeneity ^24^.

As previously mentioned, in our study, only a small percentage of the patients had intestinal symptoms, which can explain the data reported. Another factor that may be associated with the low prevalence of these protozoa was that most of the patients had CD4+ T cell counts over 200 cel/ µL (178/204; 87.3%: 95%CI: 82.7 - 91.9%). Opportunistic parasites are most common in HIV patients with a CD4+ T cell count below 200 cells/μL resulting in chronic diarrhea, extraintestinal localizations, and death ^23^^,^^24^.

Regarding helminths, only *T. trichiura* and *S. stercoralis* were detected in our study with a 0.5% prevalence each. These are soil-transmitted nematodes frequent in tropical and subtropical areas from developing countries. *Strongyloides stercoralis* is considered an opportunistic parasite in immunocompromised individuals who are at risk of developing complicated strongyloidiasis when the cell-mediated immunity is altered ^30^. Some studies have shown an increased risk of *S. stercoralis* infection in HIV-positive individuals compared to HIV-negative ones ^31^^,^^32^ and a meta-analysis of case-control studies found that the risk for HIV/AIDS patients was twice as high as that for individuals without HIV/AIDS ^30^. However, HIV infection is mainly associated with local intestinal strongyloidiasis and rarely progresses to disseminated disease, a condition with high morbidity and mortality ^30^^,^^33^, probably associated with the modulation of the immune system by HIV, which increases TH2 cytokines and decreases TH1 ones favoring intracellular microorganism infections rather than helminthic infections ^30^.

Among the risk factors evaluated in our study, only age and having pets showed a significant association with intestinal parasitic infection. Zoonotic enteric parasites are ubiquitous and remain a public health threat to humans due to their close contact with domestic and wild animals ^34^. Intestinal parasites such as *Cryptosporidium* spp., *Blastocystis*, and *Giardia* have animal hosts that facilitate their human transmission; in this sense, several studies have shown an important role of domestic animals as reservoirs in the transmission of numerous enteroparasites ^9^^,^^35^^,^^36^. In our study, all the patients evaluated were adults (over 18 years old) and, on average, the parasitized individuals were older than those non-parasitized, which was statistically significant. These differences could be related to the immunosenescence that occurs with age and the decreased response to infectious agents, i.e., bacteria, viruses, fungi, and parasites, in older people ^37^.

The introduction of the highly active antiretroviral therapy (HAART) as the main treatment for HIV has led to a reduction in infections frequency including those caused by enteroparasites and has improved the clinical and laboratory outcomes of the patients ^38^. In our study, most of the subjects were under HAART treatment, so their viremia was controlled, and we found no statistically significant association between the presence of parasites and these variables. Adherence to HAART treatment has become crucial in the clinical and public health management of HIV infection.

A cross-sectional study in HIV-infected children under antiretrovirals treatment in Cameroon determined that long-term HAART decreased the chances of intestinal parasite infection ^39^. Zorbozan, *et al.*^40^ investigated the presence of intestinal protozoa in HIV-positive patients with gastrointestinal complaints and found that the duration of antiretroviral treatment was significantly higher in non-infected patients suggesting an important role of this therapy in the reduction of the risk of intestinal parasitic infection. Taye, *et al.* also found that the magnitude of intestinal parasitic infection was slightly higher in ART-naïve HIV-positive individuals than HIV-positive patients on ART ^41^.

Our study found an intestinal parasite prevalence of 29.2% in a cohort of HIV-positive patients, the protists *Blastocystis* spp. and *E.histolytica/ dispar/moshchovskii* being the most frequent. Opportunistic protozoa like *Cryptosporidium* and *C.belli* had a low frequency, which could be related to the normal CD4+ counts of most of these patients. A strength of the study was the use of different techniques for the intestinal parasite diagnosis including the sedimentation protocol and modified agar plate, which increased the detection sensitivity for protozoa helminths and nematode larvae, respectively. Additionally, modified Ziehl Neelsen staining allowed the identification of intestinal apicomplexa including *Cryptosporidium* spp. DNA from samples of our study is available for future analysis to compare the performance of conventional and molecular techniques and give information about circulating species and genotypes contributing to the intestinal parasite molecular epidemiology in the population evaluated.

Considering that several regions of Colombia are at high risk of intestinal parasite infection, routine screening for gastrointestinal parasites in HIV patients should be implemented to contribute to their proper management. Additionally, we should emphasize that despite the appropriate virologic control, patients with HIV continue to have parasitic infections, especially in low-income countries. These infections, specifically helminths infections, can be detrimental to the human host thus increasing the susceptibility to coinfections and reducing vaccine response. On account of these data and our results, we think it is necessary to further study the factors associated with these coinfections and their effects on the chronic response to HIV.
